# Platelet-Rich Plasma Provides Superior Clinical Outcomes Without Radiologic Differences in Lateral Epicondylitis: Randomized Controlled Trial

**DOI:** 10.3390/medicina61050894

**Published:** 2025-05-14

**Authors:** Taha Kizilkurt, Ahmet Serhat Aydin, Taha Furkan Yagci, Ali Ersen, Celal Caner Ercan, Artür Salmaslioglu

**Affiliations:** 1 Department of Orthopedics and Traumatology, Medical School, Istanbul University, 34093 Istanbul, Turkey; tahafurkanyagci@gmail.com (T.F.Y.);; 2 Radiology Department, Medical School, Istanbul University, 34093 Istanbul, Turkey; cce@istanbul.edu.tr (C.C.E.); asalmaslioglu@gmail.com (A.S.)

**Keywords:** lateral epicondylitis, tennis elbow, tendonitis, platelet-rich plasma (PRP), corticosteroid, ultrasonography

## Abstract

*Background and Objectives:* Lateral epicondylitis, commonly known as tennis elbow, is a prevalent condition characterized by pain and tenderness over the lateral epicondyle. Various treatment options, including corticosteroids, platelet-rich plasma (PRP), and saline injections, are utilized, yet their comparative efficacy remains unclear. *Hypothesis:* This study hypothesizes that PRP injections result in superior functional and clinical outcomes compared to corticosteroid and saline treatments, as assessed by clinical scoring systems and radiological findings. *Materials and Methods:* The study enrolled patients aged 18 years and older with pain and tenderness over the lateral epicondyle persisting for at least three months and no prior treatment. Patients with comorbidities affecting the upper extremity were excluded. Fifty-five elbows from 50 patients were randomized into three groups (glucocorticoid, PRP, and saline). Functional outcomes were assessed using the Visual Analog Scale (VAS), Patient-Rated Tennis Elbow Evaluation (PRTEE), and Disabilities of the Arm, Shoulder, and Hand (DASH) questionnaire. Radiological evaluations included vascularity and superb microvascular imaging (SMI) indices via ultrasonography before injection and three months post-injection. *Results:* Fourteen patients were lost to follow-up, leaving 36 patients (36 elbows, 16 males and 20 females; mean age 42.4 ± 6.15 years) for analysis. The glucocorticoid group included 13 elbows, PRP group 14 elbows, and saline group 14 elbows. Baseline functional and radiological scores were similar across groups. At three months, PRP and glucocorticoid groups showed no significant differences in VAS scores (*p* = 0.7), but PRP outperformed both of the other groups in DASH and PRTEE scores, with the saline group performing the worst (*p* < 0.001). PRP consistently achieved the best outcomes at both three and six months. Radiological assessments revealed no significant group differences in vascularity or SMI indices (*p* = 0.3 and *p* = 0.2, respectively). *Conclusions:* PRP treatment demonstrated superior functional outcomes in early and mid-term evaluations compared to glucocorticoid and saline. However, ultrasonographic measures of vascularity and SMI did not correlate with functional outcomes. *Clinical Relevance:* PRP offers a promising treatment option for lateral epicondylitis, with superior functional improvements over other commonly used injections. Radiological assessments of vascularity and SMI may not reliably predict clinical outcomes.

## 1. Introduction

Lateral epicondylitis, commonly referred to as “tennis elbow”, is a prevalent condition that affects the insertion site of the forearm extensor muscles at the lateral epicondyle [1]. It occurs in approximately 1–3% of the population, with a higher prevalence in individuals aged 40 to 55 years [2]. Lateral epicondylitis predominantly affects the dominant side, particularly in individuals engaged in occupations requiring strong grip and repetitive wrist movements [3]. Additional risk factors include frequent keyboard use, overhead activities, and exposure to hand-transmitted vibrations from tools such as hammers and drills [4]. The condition is associated with several adverse effects, including functional impairment in daily and professional activities, increased psychological stress, and prolonged workplace absenteeism [5]. With the aging population, the need for effective and minimally invasive treatment modalities has become increasingly important [6]. Various treatment options for lateral epicondylitis have been described in the literature, including rest, anti-inflammatory medications, cryotherapy, bracing, botulinum toxin injections, acupuncture, corticosteroid injections, and platelet-rich plasma (PRP) injections [7,8]. However, there remains significant controversy regarding the most effective treatment approach. Despite advancements in treatment techniques, standardized protocols are lacking, leading to variability in clinical practice [9].

The self-limiting nature of lateral epicondylitis, with 80% of patients recovering within one year, may explain the wide variety of treatment options available [10]. The efficacy of PRP, corticosteroid, and placebo (saline) injections has been widely debated among researchers. PRP is commonly utilized in various fields, including wound healing, bone nonunion treatment, plastic surgery, and dermatology [11].

The use of PRP in the treatment of lateral epicondylitis is based on its theoretical ability to stimulate repair mechanisms and promote healing, similar to its effects in other tendinopathies [12]. Its anti-inflammatory properties have been demonstrated in cellular studies [13]. The literature reports that a single PRP injection can significantly improve quality of life even six months post-treatment and may be superior to corticosteroid injections [14,15].

Recent reviews and randomized controlled studies have indicated that placebo saline injections, PRP, and corticosteroid injections yield similar outcomes in the medium and long term [16,17,18].

In addition to treatment, the diagnosis and follow-up for lateral epicondylitis are equally important. Ultrasonography (USG) is particularly advantageous due to its affordability, accessibility, and ease of use [19]. Quantitative ultrasonographic measurements have shown high inter-reader reliability. A common extensor tendon cross-sectional area ≥ 32 mm^2^ and a thickness of ≥ 4.2 mm have been found to correlate with lateral epicondylitis [20]. Furthermore, increased neovascularization in patients with lateral epicondylitis has been reported to be more effectively visualized using superb microvascular imaging (SMI) compared to Doppler USG [21].

The aim of this study was to determine whether clinical and radiological changes are correlated in the mid-term following corticosteroid, PRP, and saline injections in patients with lateral epicondylitis. Based on our hypothesis, changes in clinical scores are not correlated with radiological findings. To investigate this, we sought to address the following questions:

1. Which injection treatment demonstrates greater success in the mid-term?
2. What is the mid-term radiological changes observed before and after the injection treatments?

## 2. Materials and Methods

### 2.1. Study Design and Participants

On 2 April 2021, this study (approval number 10180) was approved by the Ethics Committee and registered on ClinicalTrials.gov under the identification code NCT04875338. Between April 2021 and April 2022, individuals presenting with pain around the lateral epicondyle during wrist and finger extension against resistance, along with tenderness upon palpation, were diagnosed with lateral epicondylitis and considered for inclusion in the study [22]. Patients with symptoms lasting at least three months, aged over 18 years, and who had not received prior treatment were included in the study. A power analysis was conducted during the planning phase, and using Lehr’s formula, it was determined that a minimum of nine patients per group would be required to achieve 80% power at a 5% significance level [23].

### 2.2. Human Ethics and Consent to Participate

This study was conducted in accordance with the Declaration of Helsinki and approved by the Ethics Committee of Istanbul University Faculty of Medicine. All participants were fully informed about the study’s objectives, procedures, potential risks, and benefits. Written informed consent was obtained from each participant before enrollment. Participation was voluntary, and patients had the right to withdraw from the study at any time without consequence. Personal data were anonymized to ensure confidentiality.

**Figure 1:**
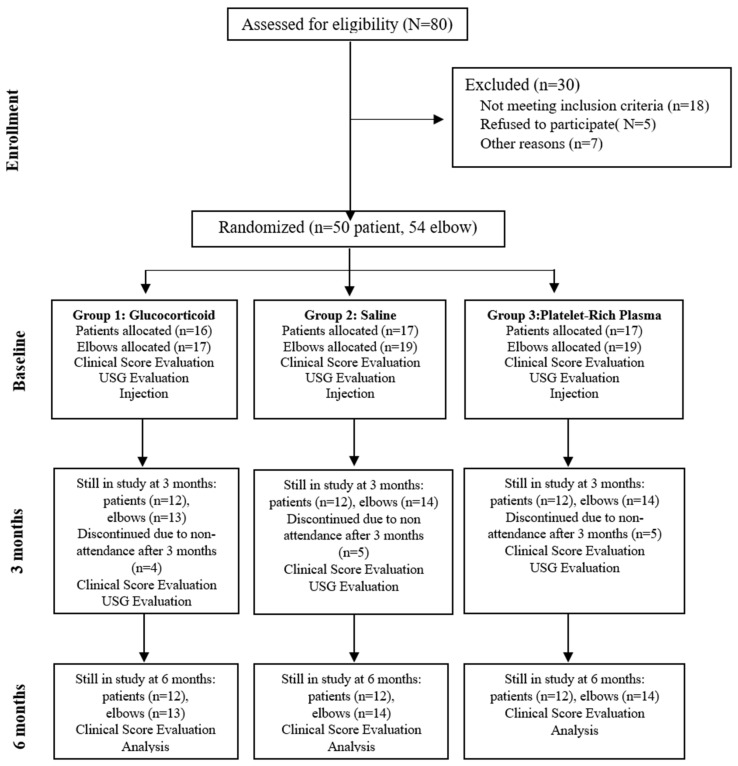
Flow diagram of patients through the study.

The study was designed as a prospective trial. Patients with elbow deformities, neurologic disorders, systemic inflammatory diseases, cervical radiculopathy, or a history of surgery on the same upper extremity were excluded. A total of 55 elbows from 50 patients meeting the inclusion criteria were enrolled in the study. Prior to treatment initiation, patients were randomized into three groups in a 1:1:1 ratio (Figure 1).

### 2.3. USG Procedure

**Figure 2:**
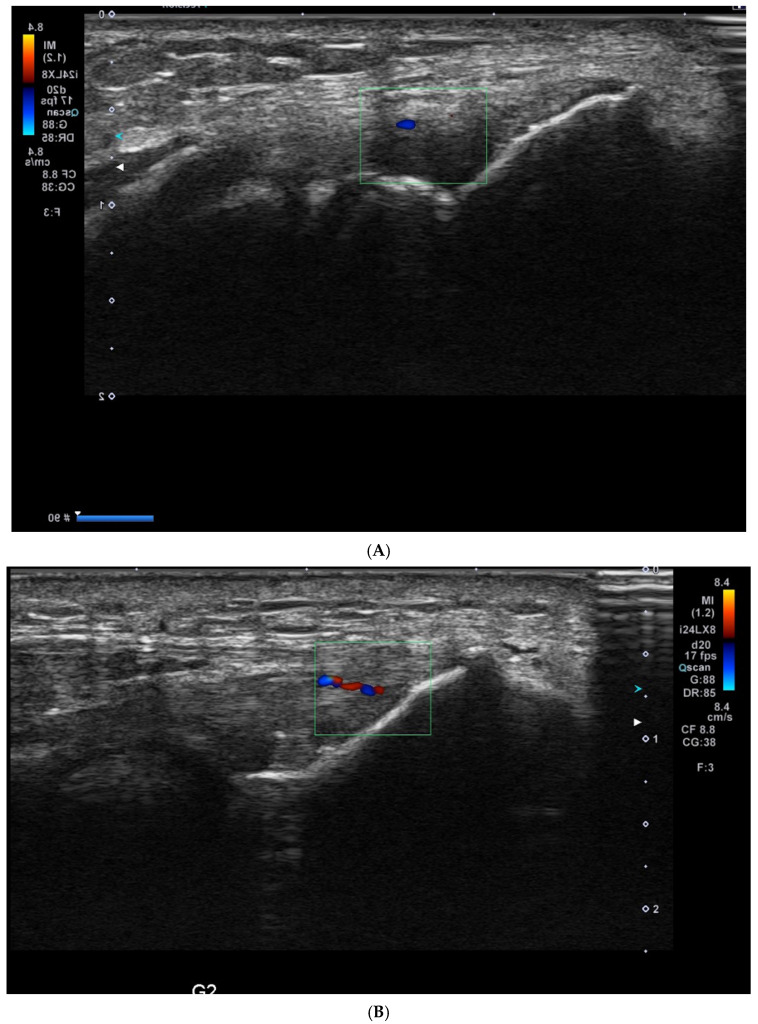

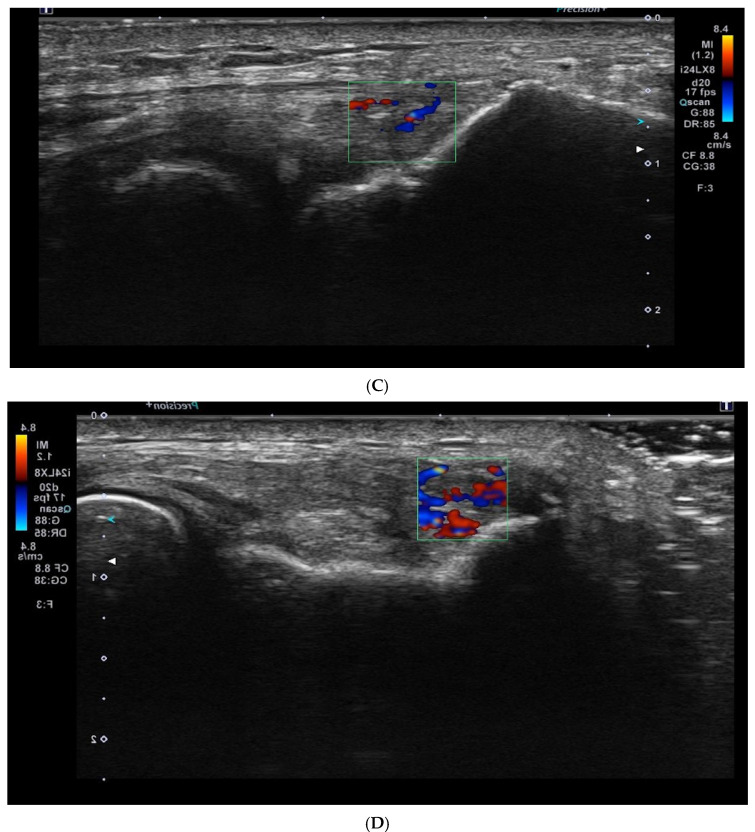
Ultrasound images illustrating vascularity grading in lateral epicondylitis using Doppler imaging: (**A**) Grade 1: single-vessel activity; (**B**) Grade 2: Doppler activity ≤ 25%; (**C**) Grade 3: Doppler activity between 25 and 50%; and (**D**) Grade 4: Doppler activity ≥ 50%.

Ultrasound (USG) evaluations were conducted jointly by two specialists following the clinical diagnosis. The physicians performing the USG were blinded to the patients’ clinical outcomes and treatment details. The USG procedure was repeated three months post-treatment. Color Doppler imaging (frame rate 10–15 Hz) and superb microvascular imaging (SMI; frame rate > 50 Hz) were performed using the Toshiba Aplio 500 USG system with a high-frequency linear array transducer. Vascular activity on color Doppler imaging was graded on a scale of 0 to 4: Grade 0 = no activity, Grade 1 = single vessel, Grade 2 = Doppler activity < 25%, Grade 3 = Doppler activity 25–50%, and Grade 4 = Doppler activity > 50% [19] (Figure 2).

**Figure 3:**
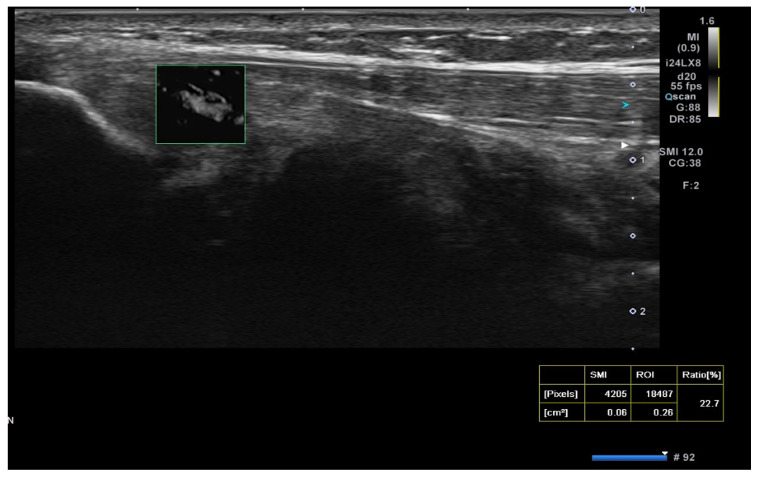
Superb microvascular imaging (SMI) in USG for lateral epicondylitis. The SMI technique enhances visualization of microvascular structures with high sensitivity, distinguishing low-velocity blood flow that may be associated with tendon inflammation and neovascularization.

Superb microvascular imaging (SMI) was assessed alongside Doppler ultrasonography (USG) as described in the literature, allowing for a more quantitative evaluation of vascularity [21] (Figure 3).

After selecting eligible patients, randomization was performed using randomization lists generated on the website random.org. Patients diagnosed bilaterally received the same treatment on both sides.

Following treatment allocation, the first group received glucocorticoid injections, the second group received saline injections, and the third group received PRP injections using the peppering technique at the point of maximal tenderness over the lateral epicondyle at the common extensor origin. Vascularity (color Doppler activity) and the superb microvascular imaging (SMI) index were assessed using ultrasonography (USG) before the injections and three months post-injection.

The study was designed as a single-blinded study. While the physician administering the treatment and the patient were aware of the treatment received, the physicians performing the ultrasonographic evaluations and clinical assessments were blinded to the treatment allocation.

Injections were administered as a single procedure. The lateral epicondyle area was disinfected with sterile iodine prior to all injections, and local anesthesia was not used. The injection volume was standardized to 2 mL for each group. For the PRP group, the CellPhi PRP CP20 (PALMED Health Product Company) kit was utilized. To prepare the PRP, 16.2 mL of the patient’s blood was drawn into a kit tube containing 1.8 mL of sodium citrate and centrifuged at 1890 G for 10 min to isolate the PRP. A total of 2 mL of the resultant PRP was injected. For the corticosteroid group, a 2 mL solution was prepared by combining 1 mL of betamethasone with 1 mL of bupivacaine hydrochloride. The post-injection protocol was consistent across all patients.

Patients were instructed to minimize the use of the affected arm following the injection and were advised to resume normal activities after 3–4 days, provided their pain was tolerable. Acetaminophen was recommended for pain management if analgesic medication was needed. Additionally, patients were advised to follow a standard stretching exercise protocol for tennis elbow [24].

Visual Analog Scale (VAS), Personalized Tennis Elbow Evaluation (PRTEE), and Disabilities of Arm, Shoulder and Hand Questionnaire (DASH) [25,26] scores were obtained before injection, after 3 months, and after 6 months following injection.

### 2.4. Statistical Analysis

Statistical analyses were conducted using SPSS version 22 (IBM Corp., Armonk, NY, USA). The normality of data distribution was assessed using the Kolmogorov–Smirnov test. To evaluate the baseline homogeneity among the study groups, a one-way analysis of variance (ANOVA) was used for continuous variables, and the chi-square test was applied for categorical variables such as gender.

Group comparisons of outcome measures (VAS, DASH, and PRTEE scores) at 3 and 6 months were performed using a one-way ANOVA. In the presence of significant differences, post-hoc pairwise comparisons were conducted using the Tukey honestly significant difference (HSD) test.

Correlations between radiological measures (vascularity score and SMI index) and clinical scores (VAS, DASH, and PRTEE) at baseline were assessed using Pearson correlation for normally distributed data and Spearman rank correlation for non-normally distributed data. A *p*-value of <0.05 was considered statistically significant.

In addition to *p*-values, Cohen’s d effect sizes were calculated to evaluate the clinical relevance of observed differences. Effect size was interpreted as small (d = 0.2), medium (d = 0.5), or large (d ≥ 0.8), according to Cohen’s conventional benchmarks. For the primary comparison between PRP and saline groups at 6 months, Cohen’s d was computed for both DASH and PRTEE scores to quantify the magnitude of clinical improvement

## 3. Results

There were no significant differences among the three groups prior to treatment in terms of age (*p* = 0.7), baseline VAS score (*p* = 0.4), baseline DASH score (*p* = 0.5), baseline PRTEE score (*p* = 0.7), vascularity score (*p* = 0.09), or SMI index (*p* = 0.6). Additionally, the chi-square test showed no significant gender differences between the groups at the start of treatment (*p* = 0.5) (Table 1). Based on these results, the groups were determined to be homogeneous before treatment.

Pearson correlation analysis was conducted to assess the relationship between the SMI index and baseline VAS, DASH, and PRTEE scores. No significant correlations were found between the SMI index and baseline VAS (*p* = 0.4), baseline DASH (*p* = 0.6), or baseline PRTEE scores (*p* = 0.4).

Spearman correlation analysis was conducted to assess the relationship between the vascularity score and baseline VAS, DASH, and PRTEE scores. No significant correlations were observed between the vascularity score and baseline VAS (*p* = 0.2), baseline DASH (*p* = 0.5), or baseline PRTEE scores (*p* = 0.2).

The results were evaluated based on the scores at 3 and 6 months. VAS, DASH, and PRTEE scores at both 3 and 6 months were compared among the three study groups. A one-way analysis of variance (ANOVA) revealed statistically significant differences across all scores between the three groups (*p* < 0.001 for all scores). Post-hoc analysis was conducted to identify the source of the differences among the groups.

No significant difference was found between the glucocorticoid and PRP groups in the VAS score evaluation at 3 months (*p* = 0.7). However, a significant difference was observed between the glucocorticoid group (4.1 ± 1.6) and the saline group (6.3 ± 2.1) (*p* = 0.02), as well as between the saline group (6.3 ± 2.1) and the PRP group (2.3 ± 2.3) (*p* < 0.001).

In the DASH score evaluation at 3 months, significant differences were observed among all groups. The glucocorticoid group (34.6 ± 13.1) showed a significant difference compared to the PRP group (19.2 ± 11.3) (*p* = 0.002) and the saline group (49.1 ± 8.1) (*p* = 0.003). Additionally, the saline group demonstrated significantly higher scores compared to the PRP group (*p* < 0.001). The PRP group had significantly lower DASH scores at 3 months compared to the other groups, indicating better functional outcomes.

For the PRTEE scores at 3 months, significant differences were observed among all groups. The glucocorticoid group (34.2 ± 9.5) demonstrated significant differences compared to the PRP group (19 ± 11.5) (*p* = 0.001) and the saline group (47.4 ± 8.5) (*p* = 0.003). Additionally, the saline group showed significantly higher scores compared to the PRP group (*p* < 0.001). The PRP group had significantly lower PRTEE scores at 3 months compared to the other groups, indicating better clinical outcomes.

At 6 months, the analysis of the VAS scores revealed no significant difference between the glucocorticoid and saline groups (*p* = 0.2). However, significant differences were observed between the glucocorticoid group (4.4 ± 1.9) and the PRP group (1.5 ± 2.2) (*p* = 0.001), as well as between the saline group (5.6 ± 1.7) and the PRP group (*p* < 0.001).

The DASH scores at 6 months showed significant differences among all groups. Comparisons revealed significant differences between the glucocorticoid group (32.6 ± 13.7) and the PRP group (13.7 ± 10.5) (*p* < 0.001), the glucocorticoid group and the saline group (45 ± 9.7) (*p* = 0.001), and the saline group and the PRP group (*p* < 0.001).

Similarly, for PRTEE scores at 6 months, significant differences were observed among all groups. Comparisons showed significant differences between the glucocorticoid group (33.2 ± 12.4) and the PRP group (14.8 ± 11.2) (*p* < 0.001), the glucocorticoid group and the saline group (44.1 ± 6.7) (*p* = 0.003), and the saline group and the PRP group (*p* < 0.001). The PRP group consistently demonstrated significantly lower PRTEE scores at 6 months compared to the other groups, indicating superior clinical outcomes (Table 2).

No statistically significant differences were found between the groups in the final vascularity scores (*p* = 0.3) or the final SMI scores (*p* = 0.2) (Table 3).

## 4. Discussion

In our study, the PRP group exhibited a marked reduction in VAS scores from 6.5 to 2.3 at 3 months and further to 1.5 at 6 months, indicating a sustained and progressive decrease in pain. These results highlight the superior and lasting analgesic effect of leukocyte-rich PRP compared to glucocorticoid and saline injections. Notably, the corticosteroid group showed a short-term improvement (VAS decreased from 6.1 to 4.1 at 3 months), followed by a slight rebound to 4.4 at 6 months, reinforcing the transient nature of corticosteroid efficacy. In contrast, PRP maintained and enhanced its therapeutic benefit over time [27]. Compared to previous studies—such as Morella et al. (VAS 7.6 to 1.5), Thanasas et al. (6.1 to 1.9), and Linnanmaki et al. (5.7 to 4.3)—our findings suggest that leukocyte-rich PRP may provide more robust and durable pain relief than leukocyte-poor formulations, warranting further investigation in standardized clinical protocols [1,17,28].

The PRTEE is a highly specific tool for evaluating lateral epicondylitis. Previous studies have reported that it is practical and valuable for assessing epicondylitis in the Turkish population [29].

In a randomized controlled study comparing injection therapies for the treatment of lateral epicondylitis, Krogh et al. reported improvements in PRTEE scores across all groups. The PRP group improved from 51 ± 4.3 to 16 ± 4.3, the corticosteroid group improved from 51.8 ± 4.3 to 13.8 ± 4.3, and the saline injection group improved from 47.1 ± 5 to 7.6 ± 4.3 [30]. In our study, PRTEE scores improved from 48 ± 8.2 to 19 ± 11.3 in the PRP group, from 46.1 ± 12.4 to 34.6 ± 13.1 in the glucocorticoid group, and from 48.9 ± 8.5 to 47.4 ± 8.5 in the saline group. Due to clinical dissatisfaction with saline injections, four patients (four elbows) withdrew from the study and received alternative treatments (two PRP and two corticosteroid injections). These patients were not included in the final analysis or the ultrasonographic (USG) examination.

In addition to statistical significance, evaluating the minimal clinically important difference (MCID) helps determine whether observed improvements are meaningful to patients. For the DASH questionnaire, the MCID has been reported to range from 10 to 15 points in upper extremity conditions, while for PRTEE, a change of 11 points or more is typically considered clinically significant [31,32]. In our study, the PRP group demonstrated a mean improvement of 34.3 points in DASH and 29.2 points in PRTEE at 6 months, both well above their respective MCID thresholds. Furthermore, we calculated Cohen’s d effect sizes for PRP versus saline at 6 months, which yielded large effect sizes (DASH: d ≈ 2.2; PRTEE: d ≈ 2.1), reinforcing the clinical and practical significance of PRP therapy. These findings suggest not only statistically but also clinically meaningful improvements in function and symptom relief with PRP treatment.

Linman et al. conducted a randomized controlled study treating patients with lateral epicondylitis using PRP, saline, and autologous blood injections. At the 3-month follow-up, DASH scores were observed to decrease from 35.6 ± 15.5 to 24 ± 19 in the PRP group and from 37.8 ± 14.8 to 27 ± 18 in the saline group [17]. However, the study by Linman et al. did not include a glucocorticoid group. In our study, DASH scores decreased from 48.3 ± 9.3 to 34.6 in the glucocorticoid group, from 50.84 ± 6.9 to 49.1 ± 8 in the saline group, and from 48 ± 5.6 to 19.2 ± 11 in the PRP group at the 3-month follow-up. Similarly, in a randomized controlled study comparing PRP and corticosteroids, Gautam et al. reported a decrease in DASH scores from 69.7 ± 6.1 to 33.6 ± 5.1 in the PRP group and from 67.5 ± 6.9 to 34.3 ± 3.3 in the corticosteroid group [33].

In both studies mentioned above, improvements in clinical scores were observed at the 3-month follow-up; however, the baseline severity of symptoms differed. In our study, the initial DASH scores were more moderate, and the 3-month results were consistent with findings reported in the literature.

Another important consideration in interpreting our results is the potential influence of the nocebo effect in the saline group. While saline injections are commonly used as a placebo comparator in injection studies, participants may perceive the absence of a biologically active substance—particularly in a single-blinded design—which could diminish their expectations of benefit [34]. This perception may negatively impact self-reported outcomes such as VAS, DASH, and PRTEE scores, independent of the actual physiological effect of the treatment [35]. In our study, four patients in the saline group withdrew early due to lack of perceived benefit, further supporting the possibility that expectancy bias contributed to the group’s poor performance. Future studies employing double-blinded designs or using active control comparators may help mitigate this limitation and provide a more robust assessment of treatment efficacy.

In our study, the follow-up of epicondylitis treatment was conducted using Doppler ultrasonography (USG) and superb microvascular imaging (SMI). We did not employ other methods, such as tendon thickness measurement or USG elastography, which have been described in the literature. Previous studies have reported that tendon thickness may vary between individuals and may not show significant changes in the early and mid-term follow-up periods [4,36].

The mean Doppler USG grades at baseline were 2.9 ± 1.1 in the PRP group, 1.9 ± 1.5 in the saline group, and 2.2 ± 1.4 in the glucocorticoid group, which decreased to 2.1 ± 1.4, 1.7 ± 1.4, and 1.4 ± 1, respectively, after treatment. These changes showed no correlation with clinical score improvements. Consequently, we utilized the SMI method, which is more sensitive and specific than Doppler USG grading for lateral epicondylitis, providing a more accurate demonstration of vascularity at the myotendinous junction [21].

In our study, the SMI technique was utilized for the first time in the follow-up after injection, differing from previous literature. The percentage change in SMI measurements was not significant among the glucocorticoid (9.8 ± 7.8 from 9.8 ± 9.3), saline (10.24 ± 8.4 from 9.3 ± 7.5), and PRP (7.06 ± 6.6 from 12.4 ± 6.9) groups.

Unlike our study, Chaundry et al. utilized contrast-enhanced ultrasonography (USG) to evaluate the effects of PRP in the treatment of lateral epicondylitis. They performed USG evaluations at 1 and 6 months post-PRP injection in six patients with an average age of 50 years and reported improvements in tendon morphology and increased vascularity between the first and sixth months. Similarly, Fredberg et al. demonstrated the vascularity-reducing effects of corticosteroids on the Achilles and patellar tendons [37]. Krogh et al. further observed a decrease in vascularity on Doppler imaging after saline and glucocorticoid injections, while an increase in vascularity was noted following PRP injections [30].

Despite its superior clinical outcomes, the use of PRP in routine care may be limited by higher costs and accessibility challenges. Corticosteroid injections remain more cost-effective and widely available, particularly in resource-limited settings. A Markov decision analysis by Dold et al. evaluated cost-effectiveness in recalcitrant lateral epicondylitis and concluded that corticosteroid injections were more cost-effective than PRP over a one-year horizon unless PRP significantly reduced recurrence or improved long-term outcomes [38]. These findings highlight the importance of balancing clinical efficacy with economic considerations when selecting injection therapies.

### Limitations

Our study was originally designed to last for one year; however, it was terminated at the 6-month mark due to the persistence of complaints in the saline treatment group and to prevent crossover between groups. Additionally, we suspect that patients’ awareness of their treatment type may have influenced their clinical scores. Furthermore, the absence of ultrasonographic (USG) guidance during injections may have impacted the results.

## 5. Conclusions

PRP injections demonstrated superior early, and mid-term clinical outcomes compared to glucocorticoid and saline treatments. In contrast, saline injections yielded the least favorable results. Although ultrasonography (USG) is widely used for diagnosis, its utility in early and mid-term follow-up appears limited. Future studies with larger cohorts and double-blind designs are needed to validate these findings and to clarify the role of imaging in monitoring treatment response.

## Figures and Tables

**Table 1:** Demographic data of the patients.

Demographics	Glucocorticoid		Saline		PRP	
	Mean	SE	Mean	SE	Mean	SE
Age	42.2	10.9	40.7	9.4	43.4	6.2
	Female	Male	Female	Male	Female	Male
Gender	7	5	7	5	6	6
Dominant Side	6		7		9	

**Table 2:** Outcome measurements and group comparisons.

	Glucocorticoid		Saline		PRP		Glucocorticoid vs. Saline	Glucocorticoid vs. PRP	Saline vs. PRP
Outcome	Mean	SE	Mean	SE	Mean	SE	*p* Value	*p* Value	*p* Value
VAS at Baseline	6.1	2.07	7.2	1.3	6.5	1.4			
PRTEE at Baseline	46.1	12.4	48.9	9.2	48.2	8.2			
DASH at Baseline	48.3	9.3	50.8	6.9	48	5.6			
VAS at 3 Months	4.1	1.6	6.3	2.1	2.3	2.3	0.02	0.7	0.001
PRTEE at 3 Months	34.2	9.5	47.4	8.5	19	11.5	0.003	0.001	0.001
DASH at 3 Months	34.6	13.1	49.1	8.1	19.2	11.3	0.003	0.002	0.001
VAS at 6 Months	4.4	1.9	5.6	1.7	1.5	2.2	0.2	0.001	0.001
PRTEE at 6 Months	34.2	9.5	47.4	8.5	19	11.5	0.003	0.001	0.001
DASH at 6 Months	32.6	13.7	47.4	8.5	13.7	10.5	0.003	0.001	0.001

**Table 3:** USG and SMI outcome measurements.

	Glucocorticoid		Saline		PRP		
Outcome	Mean	SE	Mean	SE	Mean	SE	*p* Value
US Vascularity at Baseline	2.2	1.43	1.9	1.5	2.9	1.1	0.09
SMI at Baseline	9.8	9.3	9.3	7.5	12.4	6.9	0.6
US Vascularity After Treatment	2.1	1.4	1.7	1.4	1.4	1.1	0.3
SMI After Treatment	9.8	7.8	10.2	8.4	7.06	6.6	0.2

## Data Availability

The datasets generated and analyzed during the current study are available from the corresponding author upon reasonable request.
